# ALIMUS—We are feeding! Study protocol of a multi-center, cluster-randomized controlled trial on the effects of a home garden and nutrition counseling intervention to reduce child undernutrition in rural Burkina Faso and Kenya

**DOI:** 10.1186/s13063-022-06423-5

**Published:** 2022-06-01

**Authors:** Isabel Mank, Raissa Sorgho, Fanta Zerbo, Moubassira Kagoné, Boubacar Coulibaly, John Oguso, Michael Mbata, Sammy Khagayi, Erick M. O. Muok, Ali Sié, Ina Danquah

**Affiliations:** 1 Heidelberg Institute of Global Health (HIGH), Medical Faculty and University Hospital Heidelberg, Heidelberg University, Im Neuenheimer Feld 324, 69120 Heidelberg, Germany; 2 German Institute for Development Evaluation (DEval), Bonn, Germany; 3 Centre de Recherche en Santé de Nouna (CRSN), Nouna, Burkina Faso; 4 Kenya Medical Research Institute (KEMRI), Centre for Global Health Research (CGHR), Kisumu, Kenya

**Keywords:** Evaluation, Stunting, Malaria, Bio-diversification, Behavioral change, Randomized controlled trial, Sub-Saharan Africa

## Abstract

**Background:**

Climate change heavily affects child nutritional status in sub-Saharan Africa. Agricultural and dietary diversification are promising tools to balance agricultural yield losses and nutrient deficits in crops. However, rigorous impact evaluation of such adaptation strategies is lacking. This project will determine the potential of an integrated home gardening and nutrition counseling program as one possible climate change adaptation strategy to improve child health in rural Burkina Faso and Kenya.

**Methods:**

Based on careful co-design with stakeholders and beneficiaries, we conduct a multi-center, cluster-randomized controlled trial with 2 × 600 households in North-Western Burkina Faso and in South-Eastern Kenya. We recruit households with children at the age of complementary feed introduction (6–24 months) and with access to water sources. The intervention comprises the bio-diversification of horticultural home gardens and nutritional health counseling, using the 7 Essential Nutrition Action messages by the World Health Organization. After 12-months of follow-up, we will determine the intervention effect on the primary health outcome height-for-age *z*-score, using multi-level mixed models in an intention-to-treat approach. Secondary outcomes comprise other anthropometric indices, iron and zinc status, dietary behavior, malaria indicators, and household socioeconomic status.

**Discussion:**

This project will establish the potential of a home gardening and nutrition counseling program to counteract climate change-related quantitative and qualitative agricultural losses, thereby improving the nutritional status among young children in rural sub-Saharan Africa.

**Trial registration:**

German Clinical Trials Register (DRKS) DRKS00019076. Registered on 27 July 2021.

## Administrative information

Note: the numbers in curly brackets in this protocol refer to SPIRIT checklist item numbers. The order of the items has been modified to group similar items (see http://www.equator-network.org/reporting-guidelines/spirit-2013-statement-defining-standard-protocol-items-for-clinical-trials/).

**Table:** 

Title {1}	ALIMUS – We are feeding! Study protocol of a multi-center, cluster-randomized controlled trial on the effects of a home garden and nutrition counselling intervention to reduce child undernutrition in rural Burkina Faso and Kenya
Trial registration {2a and 2b}.	DRKS00019076
Protocol version {3}	19.03.2022, Version 1
Funding {4}	The ALIMUS trial in Burkina Faso is funded by the Robert Bosch Foundation within the “Robert Bosch Junior Professorship 2019 for Research into the Sustainable Use of Natural Resources” (Reference: D10053331). The ALIMUS trial in Kenya is funded by the German Research Foundation (DFG) within the DFG Research Unit “Climate change and health in sub-Saharan Africa” (Reference: D10041684).
Author details {5a}	Heidelberg Institute of Global Health (HIGH), Medical Faculty and University Hospital Heidelberg, Heidelberg University, Heidelberg, Germany German Institute for Development Evaluation (DEval), Bonn, Germany Centre de Recherche en Santé de Nouna (CRSN), Nouna, Burkina Faso Kenya Medical Research Institute (KEMRI), Centre for Global Health Research, Kisumu, Kenya
Name and contact information for the trial sponsor {5b}	Robert Bosch Foundation GmbH (RBS), Heidehofstrasse 31, 70184 Stuttgart
Role of sponsor {5c}	The funders had and will have no role in study design; collection, management, analysis, and interpretation of data; writing of the manuscript; and the decision to submit the manuscript for publication. The funders do not have ultimate authority over any of these aforementioned activities.

## Introduction

### Background and rationale {6a}

Climate change is likely to accelerate the prevalence of undernutrition among children in rural sub-Saharan Africa [1–3]. Today, chronic protein-energy undernutrition, defined as stunting, is seen among 32% of children aged < 5 years in West Africa and among 39% in East Africa [4]. Also, about one third of this vulnerable age group shows deficiencies in iron and zinc, respectively [5, 6].

Climate change is defined as “a change in the state of the climate that can be identified (e.g., by using statistical tests) by changes in the mean and/or the variability of its properties and that persists for an extended period, typically decades or longer” [7]. It is driven by greenhouse gas emissions, including fluorinated gasses, nitrous oxide (N_2_O), methane (CH_4_), and to a large proportion, carbon dioxide (CO_2_) [8]. Climate change manifests in sub-Saharan Africa through increased temperatures, higher rainfall variability, and more frequent weather extremes [9].

There are several biophysical and socio-economic pathways by which climate change impacts child undernutrition [10]. Primarily, climate change affects the nutritional status of children by compromising the availability, access, utilization, and stability of food supply [11]. Beyond the impacts on agricultural yields, increased atmospheric CO_2_ levels likely alter the content of essential nutrients in crops. As demonstrated by CO_2_ enrichment experiments in chambers and under open-field conditions (FACE), specific grains and tubers experience 7–17% protein losses and 3–11% reductions in zinc and iron [12, 13]. Based on the current trajectory of CO_2_ emissions consistent with achieving 550 parts per million (ppm) by roughly 2050, projections indicate that elevated atmospheric CO_2_ will cause an additional 122 million people to be protein deficient and an additional 175 million people to be zinc deficient [14]. These CO_2_ trends will also translate into > 4% of dietary iron losses for 1.4 billion people at increased risk of anemia worldwide, mainly women of childbearing age and children aged 6–59 months [14].

Diversifying the agricultural production of smallholder subsistence households constitutes one promising strategy to counteract the quantitative and qualitative harvest losses, thereby improving dietary diversity and nutritional status [15]. We define smallholder subsistence farmers as rural households with agricultural land use (max. 2 hectares (ha)), who rely on low-input and manual labor to produce the majority of their food [16, 17]. Subsistence farming families are characterized by low adaptive capacities and, therefore, require autonomous and planned adaptation to consolidate their food and nutrient supply [17, 18]. Indeed, agricultural and behavioral strategies are the main efforts in low- and middle-income countries (LMICs) to cope with the already existing impacts of climate change on human health [19]. Home gardening and nutrition counseling projects have been implemented to improve dietary diversity and child nutritional status in West Africa [20] and East Africa [21]. However, among 99 systematically reviewed studies on climate change adaptation in LMICs, only two have performed a priori evaluations of the responses to such adaptation strategies [19].

### Objectives {7}

The study title ALIMUS literately means “We are feeding.” The major goal of ALIMUS is to determine the effects of an integrated home gardening and nutrition counseling program on child nutritional status in rural Burkina Faso and Kenya. The specific objectives are to (i) establish the intervention effects on the primary outcome height-for-age *z*-score (HAZ) as a measure of chronic protein-energy undernutrition; (ii) determine the effects on secondary outcomes, including other anthropometric measures of undernutrition, iron and zinc status, dietary behavior, clinical malaria, and household socio-economic status; and (iii) identify the contributions of underlying impact pathways, comprising knowledge gains in horticultural practices and child feeding, and increased production of nutrient-rich fruits and vegetables.

**Fig. 1:**
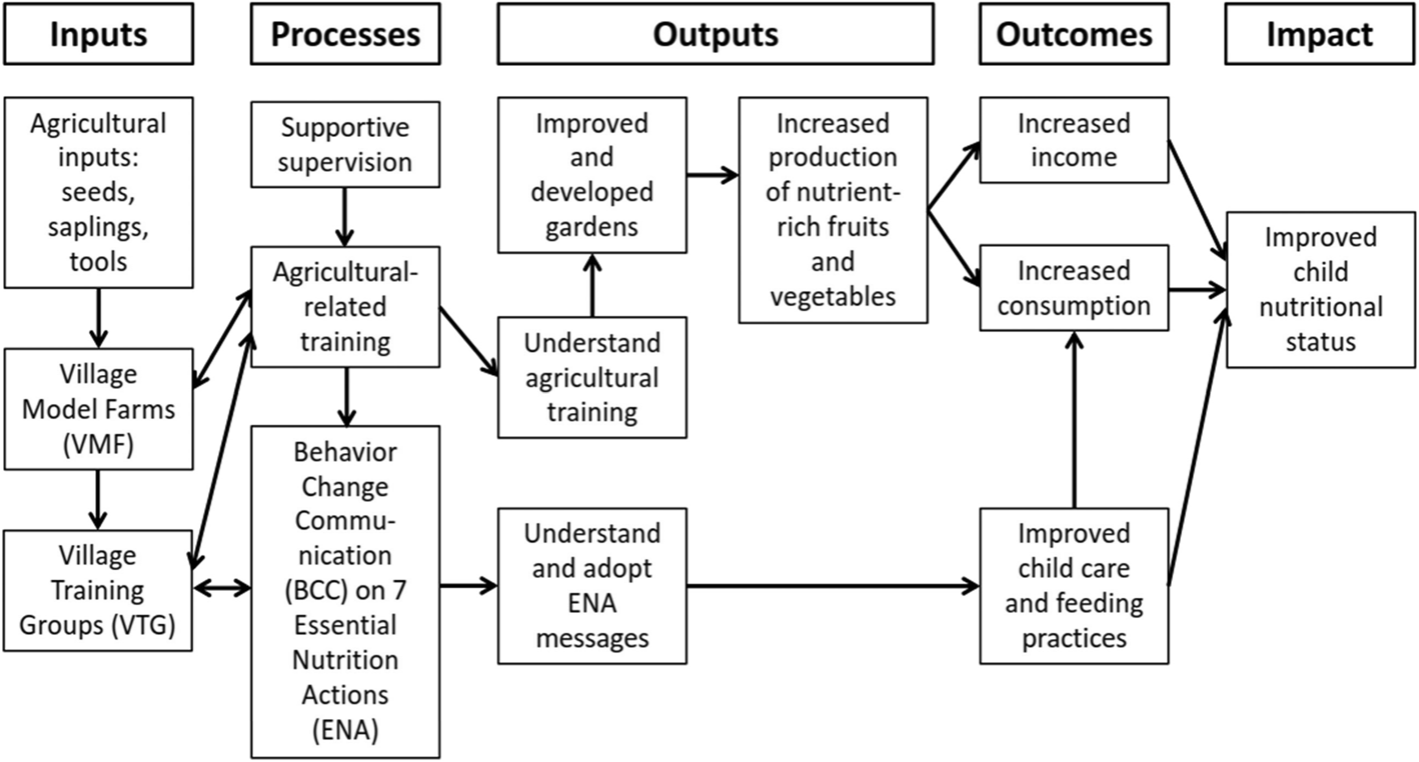
Impact pathways of the integrated agricultural bio-diversification and nutrition counseling program, modified from Helen Keller International’s Homestead Food Production Program 2013

A similar agricultural bio-diversification and nutrition counseling program was previously implemented by the non-governmental organization (NGO) Helen Keller International (HKI). This project was conducted and evaluated in Southern Burkina Faso [20, 22], but not in Kenya. Originally, HKI’s program aimed at improving mothers’ nutritional outcomes and empowerment through a set of agricultural production and nutrition interventions. The HKI program targeted mothers with children aged 3 to 12 months at baseline [23]. In contrast, ALIMUS targets the age when complementary foods are introduced to children (6 to 24 months). This acknowledges the age when stunting starts to develop among children, and accounts for the common practice of child fostering, meaning children being raised in households of relatives. The theory of change relies on three primary impact pathways (Fig. 1) to support the achievement of a healthy status of climate-sensitive nutrients among young children. These impact pathways are:

1. Increased production of climate-resilient horticultural crops and diversification of crops for increased consumption of these foods;
2. Increased knowledge related to the importance of agricultural diversification for the resilience of local crops and, thus, food and nutrient security for healthy families, thereby increasing the adoption of optimal practices; and
3. Increased output of the horticultural foods to increase income.

### Trial design {8}

The ALIMUS study is conducted as a multi-center, cluster-randomized controlled trial (RCT) with 300 households in the control group and 300 households in the intervention group at each study site. We perform baseline examinations of children at the age of complementary feed introduction (6–24 months) and follow-up these children for at least 1 year until the endline examinations. Baseline examinations take place in Burkina Faso between July and September 2021 and in Kenya between September and December 2021.

## Methods: participants, interventions, and outcomes

### Study setting {9}

ALIMUS will be conducted in the Nouna area of North-Western Burkina Faso and in the Siaya region of South-Eastern Kenya. We apply the multi-center, cluster-RCT within the Health and Demographic Surveillances Systems (HDSS) in Nouna, Burkina Faso and in Siaya, Kenya. Both were established in the early 1990s. The Nouna HDSS comprises of 59 contiguous villages in an area of 1775 km^2^ with a representative population of 115,000 inhabitants in 14,000 households [24, 25]. The Siaya HDSS is more population-dense; it contains 385 villages in an area of 700 km^2^ with 220,000 inhabitants in 54,869 households [26].

With regard to climatic and topological characteristics, the two study sites differ: Nouna experiences one rainy season per year (June to October), while there are two rainy seasons in Siaya (March to May and October to December). Average temperatures in Nouna (20–37 °C) are higher than those in the highlands of Siaya (17–35 °C). Concerning the population structure, both study sites are characterized by smallholder subsistence farming, which is complemented by cattle herding in the Nouna region and by fishing in the Siaya areas surrounding Lake Victoria. In North-West Burkina Faso, the major food crops are millet, sorghum, maize, peanuts, and sesame. In Western Kenya, they are maize, beans, sweet potatoes, sorghum, rice, and cassava. The typical diets in both regions are based on starchy staples and legumes [27]. Siaya and Nouna have similar proportions of child stunting, 26% vs. 21% respectively [28, 29]. The difference may be due to the high incidence of the human immunodeficiency virus (HIV) infection in Western Kenya (3/1000 person-years vs. 0.3/1000 person-years). In contrast, the prevalence of anemia is higher in Nouna (50% vs. 80%), possibly reflecting the poor micronutrient adequacy and the intense malaria transmission. In both study areas, malaria is endemic either during the rainy season (Nouna) or throughout the year (Siaya) with 30-50% of the children < 5 years being affected [30, 31].

### Eligibility criteria {10}

**Fig. 2:**
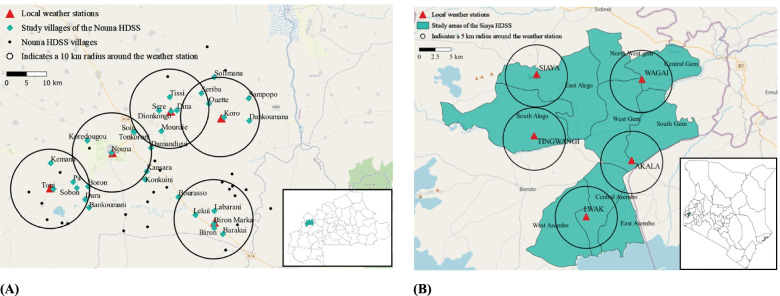
Map of the study villages in the Nouna Health and Demographic Surveillance System (HDSS), Burkina Faso (**A**) and the Kisumu HDSS, Kenya (**B**). Note: In the Kisumu HDSS, the villages are close together and, thus, separated by wider boundaries shown as polygons. All the villages located within the 5 km radii of the weather stations in the Kisumu HDSS are included in the study. In contrast, the villages in the Nouna HDSS, marked by black dots and blue diamonds, are widely spread; therefore, the weather stations cover a 10-km radii. Only some villages were randomly selected for inclusion in our study, which are marked with a blue diamond

In both study sites, the same eligibility criteria apply for participating households. A household is defined as “an independent socio-economic unit. Household members usually live in the same house or compound, pulling resources together to meet basic dietary and other vital needs under the authority of one person recognized as the head of the household” [25]. The eligibility criteria are as follows: location within a 5-km radius for Kenya and a 10-km radius for Burkina Faso of one of five local weather stations (Fig. 2), permanent residence in the HDSS area; access to at least 40 m^2^ land, access to water for implementing and sustaining a home garden, having a child at the age of supplementary feed introduction (6 to 24 months), and providing informed written consent by the caregiver.

### Who will take informed consent? {26a}

Prior to the program implementation, relevant authorities of the selected study villages (village chiefs and mayors) will be informed about the study aims and objectives by the Nouna CRSN and by the KEMRI-Kisumu. During the recruitment process, the participants will receive the participant information form, which will be read-out and explained to them. All questions will be answered by the project staff. All questionnaires, participant information forms, consent forms, and additional material will be made available in French for Burkina Faso and in English and Luo for Kenya. In addition, the interviewers will be trained to explain the content of the participant information forms in Djoula and Mooré in Burkina Faso and in Swahili in Kenya. All caregivers will give informed written consent.

### Additional consent provisions for collection and use of participant data and biological specimens {26b}

These items are part of the original consent form. This trial does not involve collecting biological specimens for storage.

### Interventions

#### Explanation for the choice of comparators {6b}

For the allocation of intervention arms, we apply simple randomization, treating the households as intervention clusters. At each site, the main data manager of the baseline survey produces a computer-generated sequence of households that are allocated to either the intervention group or the control group, applying the PPS approach. The implementation partners for home gardens (DEZLY Consulting, the Nouna Agricultural Service and CABE) and the lead dieticians for nutrition counseling use these lists of randomized households to implement the intervention components. As this is a behavior change trial, blinding the participants and or the implementation team is not possible.

All participating households in the ALIMUS trial benefit from trimonthly follow-up visits by a research study team (not Community Health Volunteers (CHVs). During these follow-up visits, the study team takes anthropometric measures and examines iron and zinc status of the target child. We also assess signs and symptoms of clinical malaria and provide diagnostic tests if a child has fever, shivers, a headache, or diarrhea. In case of sickness, the child and the caregiver are referred to the nearest health care facility for appropriate treatment. For the control group, we provide standard information on healthy feeding practices. This comprises one group counseling session on the Essential Nutrition Action (ENA) messages, but no tailored individual sessions afterwards. During the group counseling, we hand-out an information leaflet corresponding to the ENA messages.

#### Intervention description {11a}

The intervention program has two components: bio-diversification by means of home gardens and dietary diversification through behavior change communication. The intervention period will last for at least 1 year.

For the home garden component, we apply the following definition. Home gardens are small plots (< 40 m^2^) located near the house for growing vegetables and/or fruits, primarily for the household’s own food consumption. For home garden implementation, we collaborate with three experienced local partner organizations. In Burkina Faso, we work with DEZLY Consulting, a consulting service organization (CSO), and the Nouna Agricultural Service, and in Kenya, we collaborate with the NGO Centre for African Bio-Entrepreneurship (CABE). The intervention activities by partner organizations comprise sensitization of community members, distributing inputs, and providing training to garden leaders. These garden leaders come from the participating communities with one garden leader being responsible for approximately 20 beneficiary households. They have experience in home gardening, are respected members of their communities, have independent means of transportation, and are committed to working as volunteers in this project. In two phases, garden leaders are trained by DEZLY Consulting and the Nouna Agricultural Service in Burkina Faso and by CABE in Kenya on theoretical and practical aspects of home gardening. The first phase (months 1 to 3) focuses on the establishment of the home gardens, while the second phase (months 9 to 12) concentrates on the long-term viability of the gardens and a potential market access through garden produce surplus. The training elements comprise the selection of garden types and horticultural crops, marking and fencing the gardens, seed production and multiplication, methods of manure creation and composting, soil preparation, weeding and plant protection, harvesting, and food storage. We purposefully employ only climate-friendly and durable inputs and processes, such as local products for fencing and construction, participatory selection of indigenous crops that meet the beneficiaries’ preferences and are not genetically engineered. We also exclusively collaborate with regional seed providers and use organic pesticides and fertilizers. For refresher trainings and trouble-shooting, two extension officers per study site will be appointed by DEZLY Consulting in collaboration with the local Agricultural Service in Burkina Faso, and by CABE in Kenya.

In regard to the behavior change communication component, at each study site, one formally trained dietician is responsible for training and supervising CHVs. Each CHV provides nutrition counseling to about 20 households at each study site. CHVs are members of their communities appointed by the local authorities of the Ministry of Health. They have minimal training in maternal and child health or nursing and are of the same ethnic background as the ALIMUS beneficiaries. Half of the CHVs are males. The content of the nutrition counseling is sourced from the 7 ENA messages by the World Health Organization (WHO) [32]. These ENA messages are implemented in the form of the Infant and Young Child Feeding (IYCF) Cards by the Ministries of Health in Burkina Faso and Kenya. The 7 ENA messages comprise the following: (1) children’s nutritional needs (calories, protein, micronutrients), (2) breastfeeding practices, (3) complementary feeding practices, (4) nutritional care for sick and severely malnourished children, (5) prevention and control of anemia (zinc, iron), (6) prevention and control of vitamin A deficiency, and (7) prevention and control of iodine deficiency.

Nutrition counseling is provided to the beneficiaries in group sessions at the beginning of the intervention and during individual sessions in the beneficiaries’ household every 2 months. Just as the different IYCF Cards target different age groups, the individual nutrition counseling sessions are also tailored to the feeding stages of the target child.

#### Criteria for discontinuing or modifying allocated interventions {11b}

The project team closely collaborates with the local Ministries of Agriculture and Ministries of Health. In case we will observe clinically relevant improvements in the nutritional status after 1 year of the intervention, we will seek their support for offering the program to the control households, too.

#### Strategies to improve adherence to interventions {11c}

Adherence to the intervention will be supported through regular contacts to the beneficiaries during training sessions on home gardening and individual nutrition counseling. We will closely monitor the uptake of inputs such as seeds and garden tools, as well as the participation in all sessions.

#### Relevant concomitant care permitted or prohibited during the trial {11d}

No concomitant care prohibited.

#### Provisions for post-trial care {30}

No provision of post-trial care.

#### Outcomes {12}

The ALIMUS trial targets as the primary outcome the height-for-age z-score (HAZ) of the target children after 12 months of follow-up according to the 2006 WHO Child Growth Standards [33]. Secondary outcomes comprise additional anthropometric *z*-scores (weight-for-height (WHZ) and weight-for-age (WAZ)) after the 12 months intervention period; measures of dietary behavior at endline, including energy intake, consumption of macronutrients and micronutrients, food group intakes, indices of dietary diversity, and exploratory dietary patterns; the nutritional status of iron and zinc measured by hand-held spectrometry at endline; the incidence of clinical malaria; and the socio-economic status of the household.

#### Participant timeline {13}

At both study sites, each participant is followed-up for at least 1 year, with a possible extension of an additional year (Table 1). After community sensitization, eligibility screening and consent taking, we perform baseline examinations over the course of 3 months. We then randomize households and allocate them to either the intervention group (*n* = 300 at each site) or the control group (*n* = 300 at each site). In groups of 20 beneficiaries, the intervention group participants receive trainings in setting up their gardens by their respective garden leader and counseling sessions on healthy child feeding practices by CHVs. Every 2 months, garden leaders and CHVs actively visit the intervention households for guidance on home garden practices and individual dietary counseling. In 3 month intervals, we integrate anthropometric measurements and assessments of micronutrient status and clinical malaria into this schedule. After the first 6 months of the intervention, refresher trainings of the beneficiaries for home garden practices are conducted in the form of group sessions. After another 6 months (1 year since start), we conduct endline examinations for the primary and secondary outcomes among all study participants. In addition, we monitor inputs and outputs according to the three major impact pathways throughout the intervention phase.

**Table 1:** Activity timeline for the ALIMUS trial in rural Burkina Faso and Kenya

Time point	2021						2022												2023			
	Q3			Q4			Q1			Q2			Q3			Q4			Q1			Q2
**Enrolment**																						
Eligibility screening of study participants	X	X																				
Community sensitization and informed consent	X	X																				
Recruitment of study participants			X	X	X																	
Randomization and allocation					X																	
**Intervention**																						
Training of garden leaders and CHVs			X	X	X																	
Nutrition counseling																						
Group counseling (1 session only)							X	X														
Bi-monthly individual counseling sessions^a^									X	X	X	X	X	X	X	X	X	X				
Home garden training^a^																						
Organic pesticide and manure production							X	X							X							
Composting and nursery establishment							X	X							X							
Land preparation, construction and planting								X	X							X						
Weeding and plant protection									X	X						X						
Harvesting, value addition and storage										X	X						X					
Training on maintenance of home gardens												X	X	X								
**Monitoring**																						
Input monitoring (cont.)		X		X			X			X			X			X			X			
Knowledge gains																			X			
Species richness and garden yields (cont.)							X			X			X			X						
**Assessments**																						
Interviews on diet, demographics, socio-economic status		X		X	X														X	X	X	
Anthropometric examinations		X		X	X														X	X	X	
Micronutrient measurements, 3-monthly^b^									X			X			X			X			X	
Malaria assessments, 3-monthly^b^									X			X			X			X			X	
**Evaluation**																						
Cross-sectional baseline analysis				X			X															
Intention-to-treat and differences-in-differences analyses																				X	X	X

^a^Only for the intervention groups

^b^Only in Kenya

#### Sample size {14}

In order to identify the required sample size for this RCT, we referred to the parallel group- or cluster-randomized trials (GRT) Sample Size Calculator of the USA National Institutes of Health (NIH) (https://researchmethodsresources.nih.gov/). The sample size was calculated based on the primary outcome child HAZ, assuming a group difference in mean HAZ ranging between 0.18 to 0.25. At a significance level of 0.05, a statistical power of 80%, an intra-class correlation coefficient (ICC) of 0.05, and with 2 children per household (=cluster), the sample size ranges from 135 households (difference in mean HAZ of 0.25) to 260 households (difference in mean HAZ of 0.18). Therefore, at each study site, we set the sample size to 300 households in the intervention group and 300 households in the control group, accounting for potential attrition and loss to follow-up. This sample size will allow the detection of a difference in the incidence of clinical malaria of 0.16 standard deviation (SD), under the same assumptions.

#### Recruitment {15}

The participants will be recruited within the two existing HDSS in Nouna, Burkina Faso, and in Siaya, Kenya. The recruitments will be conducted by the Health Research Center (CRSN) in Nouna and by the Kenya Medical Research Institute (KEMRI)-Kisumu. These institutions have long-standing expertise in conducting demographic and health surveys in the target populations, including community sensitization and data collection. For each study site, we will establish a sampling frame of 2000 households with children below 5 years of age and within a 5- or 10-km radius of local weather stations, respective of study site. These households will be selected with a probability proportional to population size (PPS), according to the latest census. Of these, we will recruit 600 households at each study site with children who fulfill the eligibility criteria.

### Assignment of interventions: allocation

#### Sequence generation {16a}

At each site, the main data manager of the baseline survey will produce a computer-generated sequence of households stratified by the 5 weather stations. Using PPS, the households will be randomized into either the intervention group or the control group.

#### Concealment mechanism {16b}

Study beneficiaries are selected through randomization via Stata using computer-generated random numbers. There is no human involvement, and the process is fully concealed from both study investigators and prospective participants during baseline measurements and until the study arm is assigned.

#### Implementation {16c}

Based on the randomization, the field supervisor will allocate the households to the intervention groups. The study group will be revealed at the same time to the participating households, the implementation team, and the field agents.

### Assignment of interventions: blinding

#### Who will be blinded {17a}

As this is a behavior change intervention, neither blinding of the participants nor of the implementation team is feasible. The data analysts will be blinded.

#### Procedure for unblinding if needed {17b}

The study design is openly labeled, and so, unblinding will not be possible.

### Data collection and management

#### Plans for assessment and collection of outcomes {18a}

Two quantitative surveys are conducted at baseline and endline of the ALIMUS trial. These surveys comprise questionnaire-based interviews on demographic and socioeconomic characteristics, medical history, dietary information, anthropometric measurements, micronutrient status through hand-held spectrometry, body temperature, and laboratory measurements for hemoglobin concentration (Hb) and malaria diagnosis. All project personnel are thoroughly trained and work according to Standard Operating Procedures (SOPs). The study team consists of field agents who conduct the questionnaire-based interviews and anthropometric examinations; laboratory technicians who perform the blood sample collection, body temperature and Hb measurements, hand-held spectrometry and malaria diagnosis; and a monitoring team who document monetary and opportunity costs of the intervention, knowledge gain, training and counseling activities, and garden yields. The data collection instruments and variables measured are presented in Table 2.

**Table 2:** Variables measured and data collection instruments in the ALIMUS trial

Module	Measure/variable	Questionnaire/instrument
**Demographic**	Age (months), sex (male/female), mother’s age (years), mother’s ethnicity (categorical), mother’s religion (categorical)	Questionnaire
**Socioeconomic**	1. Mother’s education (categorical), mother’s occupation (categorical), mother’s marital status (categorical) 2. Number of people in the household (continuous) 3. Type of housing (roof, wall, ground), access to and type of water source (categorical), access to and type of toilet and electricity (categorical), availability of 14 household assets (yes/no), availability of 9 animals (yes/no) 4. Main source and amount of income (categorical and continuous), main type and amount of household expenditures (categorical and continuous)	1. Questionnaire 2. Questionnaire 3. Questionnaire 4. Questionnaire
**Diet**	1. Breastfeeding (starting age, ending age) 2. Complementary feeding (starting age) 3. Usual food intake	1. Questionnaire 2. Questionnaire 3. African-specific Food Propensity Questionnaire (AFPQ)
**Nutritional status**	1. Anthropometry [length/height (cm), weight (kg), mid-upper arm circumference (cm)] 2. Food insecurity 3. Hemoglobin concentration (Hb) 4. Functional status of iron and zinc	1. Measuring board SECA 417; stadiometer SECA 213; weighing scale SECA 878 2. Household Food Insecurity Access Scale (HFIAS); Household Hunger Scale (HHS) [34, 35] 3. HemoCue Hb 201+, HemoCue, Germany 4. Hand-held spectrometer (Zell-Check®, 2019)
**Malaria**	1. Body temperature (°C) 2. Self-reported history of fever in the past 48 h (yes/no) 3. Self-reported symptoms of malaria in the past 48 h (yes/no) 4. Malaria parasites (yes/no); parasite count (/μL)	1. Axillary measurement 2. Questionnaire 3. Questionnaire 4. Thick blood film and microscopy
**Process monitoring**	1. Inputs to home gardens and nutrition counseling 2. Outputs from home gardens 3. Knowledge gain from home garden training and nutrition counseling	1. Assets and activity tracking cards 2. Garden diary 3. Qualitative interview guides

The questionnaire-based modules were previously applied in a cohort study in the Nouna HDSS area [27] and are adapted to the purposes of the ALIMUS project. We assess the usual dietary intake among children using the African Food Propensity Questionnaire (AFPQ), which is a modified version of the Ghana-Food Propensity Questionnaire [36]. It queries the usual intake frequencies of 134 food groups in pre-defined portion sizes over the past 6 months. We use the West African Food Composition Table [37] and the Kenya Food Composition Tables [38] to translate food group intakes (g/d) into energy (kcal/d), macronutrients (energy), and micronutrients (mg/d).

During physical examinations, we obtain all anthropometric measurements in light clothes. Length and height are measured to the nearest cm and weight to the nearest 100 g. For children who are < 85 cm, recumbent length is obtained. For mothers and for children who can stand alone and are ≥ 85 cm, we measure standing height. Body weight is measured on a mother-and-child weighing scale. We measure body temperature of the children using an automated forehead thermometer. Hand-held cutaneous spectroscopy is used to determine the functional status of zinc and iron of the children. This laser spectroscopic technique converts inelastically scattered light from human skin into the tissue content of micronutrient compounds [39].

For laboratory examinations, we collect capillary blood samples of children. Hb is measured by hand-held photometer, and malaria parasites are microscopically identified on Giemsa-stained (4%, 30 min, pH 7.2) thick blood films. We calculate parasite density by examining microscopy fields corresponding to 400 white blood cells (WBCs), assuming an average WBC count of 8000/μL. Asymptomatic malaria is defined as the absence of fever (< 37.5 °C) and the presence of any Plasmodium spc.; clinical malaria denotes any parasite density plus fever (≥ 37.5 °C) or a history of fever within the last 48 h; and severe malaria is defined according to WHO criteria [40].

For the process monitoring, we employ tracking cards to document project inputs and activities, including equipment and seeds, garden trainings, and nutrition counseling sessions. In addition, the extension officers record the horticultural outputs in garden diaries. We conduct in-depth interviews and apply a deductive analysis strategy using pre-defined knowledge categories for home gardening and child feeding practices.

#### Plans to promote participant retention and complete follow-up {18b}

Adherence to the intervention will be supported through regular contacts to the beneficiaries during training sessions on home gardening and individual nutrition counseling. We will closely monitor the uptake of inputs such as seeds and garden tools, as well as the participation in all sessions.

#### Data management {19}

The data will be collected on tablets using the data entry software SurveySolutions (version 21.09, World Bank). The data will be checked daily by the field supervisors and weekly by the principal investigator. We ensure high data quality through regular de-briefings on the data collection and data entry procedures. The collected data will be stored securely as password-protected files (encrypted storage devices) and will be ultimately stored on the institutional cloud server at the Heidelberg Institute of Global Health (HIGH), Germany.

#### Confidentiality {27}

We ensure complete confidentiality of the data. Informed consent forms, laboratory books, and other participant-related documents will be safely stored during study conduct at the HDSS research centers in Burkina Faso and Kenya. All data and samples will be pseudonymized for analysis. The research data will be stored securely and password-protected throughout the data collection and processing stages. A digital exchange platform (secured and encrypted) at the Heidelberg University Computing Center will be used for the ALIMUS trial. All data collected will be stored in Microsoft-SQL databases that have been designed based on the Reference Demographic Surveillance Data Model, facilitating productive cross-collaboration efforts. According to the rules of good scientific practice, research data of this project will be archived for at least 10 years.

#### Plans for collection, laboratory evaluation, and storage of biological specimens for genetic or molecular analysis in this trial/future use {33}

No genetic or molecular analysis of biological material is planned in this trial.

### Statistical methods

#### Statistical methods for primary and secondary outcomes {20a}

Demographic, socio-economic, and nutritional and dietary as well as clinical characteristics of the study population will be presented as means and SDs for continuous normally distributed variables, as medians and interquartile ranges (IQRs) for skewed data, and as percentages and counts (N) for categorical data. We will use parametric hypothesis tests to compare cross-sectional baseline characteristics between the control and the intervention groups on both levels, namely households and participants.

For the characterization of dietary practices, we will employ exploratory and hypothesis-based dietary pattern analyses. These comprise principal component analysis (PCA) for the data-driven dietary patterns and the construction of diet quality scores. The latter includes the dietary diversity score (DDS), the food variety score (FVS), the minimum dietary diversity (MDD), the minimum meal frequency (MMF), the minimum acceptable diet (MAD), and the mean adequacy ratios (MAR).

The intervention effects will be determined using an intention-to-treat approach, and will be stratified by study site. We will model the primary outcome HAZ as the dependent variable and the intervention group as the independent variable in a multi-level linear regression analysis in order to obtain group-differences in mean HAZ at the end of the intervention period. The same regression analysis will be applied for all other continuous outcome variables measured at endline. For categorical outcome variables, we will use Cox regression; for the incidence of clinical malaria, we will employ negative binomial regression. We will adjust for potential imbalances in the baseline characteristics. In addition to adjustments, we will perform difference-in-differences (DID) analyses to control for unmeasured confounding. These DID estimates compare the change in outcome variables from endline to baseline examinations between the intervention and the control group [41].

For the budget impact analysis of the ALIMUS trial, we adhere to the Consolidated Health Economic Evaluation Reporting Standards (CHEERS) Statement [42] and will use the activity-based costing (ABC) approach.

#### Interim analyses {21b}

No interim analyses will be conducted. The results will only be analyzed at the end of the intervention (after 1 year).

#### Methods for additional analyses (e.g., subgroup analyses) {20b}

In addition to adjustments, we will perform difference-in-difference (DID) analyses to control for unmeasured confounding. These DID estimates compare the change in outcome variables from endline to baseline examinations between the intervention and the control group [41]. Also, we will conduct sub-group analysis according to age group of the children and by location of the weather station.

#### Methods in analysis to handle protocol non-adherence and any statistical methods to handle missing data {20c}

We will conduct per protocol analysis and compare it the intention-to-treat analysis.

#### Plans to give access to the full protocol, participant level-data, and statistical code {31c}

The data can be made available by the principle investigators for research purposes only, upon an agreed analysis proposal, and after signing a data transfer agreement.

### Oversight and monitoring

#### Composition of the coordinating center and trial steering committee {5d}

This is a multi-center study designed and coordinated by three institutions: HIGH, Heidelberg University, Heidelberg, Germany; CRSN, Nouna, Burkina Faso; and KEMRI, Kisumu, Kenya. The trials are performed in the Nouna HDSS area, Burkina Faso, and in the Siaya HDSS area, Kenya. Day-to-day support for the trial is provided by one principal nutrition counselor and two home garden officers, respectively. They run daily debriefings with CHVs as well as with garden leaders. The principal nutrition counselors and the home garden officers are also the contact persons for the on-site finance officers of the local partner organizations. The finance officers at CABE and DEZLY are responsible for procurement of inputs and equipment for the home garden component, while the finance officers at CRSN and KEMRI take care of the procurement of research equipment and consumables and the payment of research personnel. The trial steering committee consists of three investigators, representing one of the three research institutions as well as two coordinating scientists, who virtually meet in bi-weekly intervals for overall management and trouble-shooting.

#### Composition of the data monitoring committee, its role and reporting structure {21a}

A formal data monitoring committee (DMC) was not established as this is a low-risk intervention. However, each of the three research institutions allocates one independent data manager. This data manager will conduct plausibility checks, data cleaning, and analysis. The sponsors play no role in data management, analysis, and interpretation of findings. Thus, the data managers have no competing interests. During bi-weekly meetings, data managers will report their observations from plausibility checks and interim analysis to the steering committee.

#### Adverse event reporting and harms {22}

The implementation teams at each study site are equipped with monitoring tools to document referrals to the nearest health facility and the reasons for referral. We also capture reasons for lost to follow-up, including deaths.

#### Frequency and plans for auditing trial conduct {23}

The steering committee and informal data monitoring team meet online bi-weekly to review the conduct of the trial. This includes the review of adherence to intervention components, logistical aspects, feedback from beneficiary households, completeness and quality of collected data, and adherence to milestones. Also, regular exchange about the trial progress is assured via phone calls and short messages.

#### Plans for communicating important protocol amendments to relevant parties (e.g., trial participants, ethical committees) {25}

Any modifications to the study protocol, which may impact on the conduct of the study, potential benefit of the study participants or participant safety, including changes of study objectives, study design, participant population, sample size, study procedures, or essential administrative aspects, will require a formal amendment to the protocol. Amendments will be agreed upon by the principal study investigators and approved by the Ethics Committees in Heidelberg, Nouna, and Kisumu prior to implementation.

#### Dissemination plans {31a}

Regular updates of the ALIMUS project will be presented on the project website www.cch-africa.de/climate-sensitive-nutrients/. Results of the ALIMUS trial will be published in peer-reviewed high-ranked journals, disseminated via international conferences, shared in educational seminars, and reported to relevant stakeholders. The study also aims to reach the policy community first through engaging with local government representatives, civil society organizations, and community leaders in Nouna and Siaya. In addition, we will exchange with international actors in the nutrition, agriculture, energy and climate sectors, and institutions dealing with family affairs and education.

## Discussion

The ALIMUS trial will contribute to the scarce literature on the evaluation of climate change adaptation projects in sub-Saharan Africa. In two climatically distinct areas in North-Western Burkina Faso and South-Eastern Kenya, we carefully co-design integrated agro-biodiversification and nutrition programs for counteracting the visible impacts of climate change on food quantity and quality, which mainly harm the vulnerable population group of under-fives. Rigorous impact evaluation for nutrition and health outcomes, garden yields, and the household economic situation are central to the ALIMUS trial. In addition, stringent monitoring of project inputs and outputs ensure rigorous project evaluation along three major impact pathways. The concrete effect measures of nutrition and health as well as the budget impact analysis will contribute to discussions about national climate change adaptation strategies.

### Trial status

As of January 2022, beneficiary recruitment, baseline examinations, randomization, and intervention allocation have been completed. Research staff, garden leaders, and nutrition counselors were thoroughly trained. The data collection is planned to be completed by March 2023. This is protocol version 1 as of 19 March 2022. A submission of the study protocol prior to the end of recruitment was not possible due to the multi-center nature of this trial. The COVID-19 pandemic caused delays in the conduct of the study, wherefore the trial initiation was slightly delayed in Kenya and the intervention implementation had to be postponed to a later stage than planned in Burkina Faso.

## Data Availability

Reports of training sessions will be published on www.cch-africa.de/news/. Otherwise, pseudonymized data and material can be made available to third parties upon reasonable, written request to the principal investigators (PIs) and for research purposes only. Together with the ethical review boards, the PIs will review any requests for access to data and materials on the background of approved research questions and objectives.

## Abbreviations

ABC: Activity-based costing
CABE: Centre for African Bio-Entrepreneurship
AFPQ: African Food Propensity Questionnaire
CGHR: Centre for Global Health Research
CH_4_: Methane
CHEERS: Consolidated Health Economic Evaluation Reporting Standards
CHV: Community Health Volunteer
CO_2_: Carbon dioxide
cRCT: Cluster-randomized controlled trial
CRSN: Centre de Recherche en Santé de Nouna
CSO: Consulting service organization
DDS: Dietary Diversity Score
DEval: German Institute for Development Evaluation
DFG: German Research Foundation
DID: Difference-in-differences
ENA: Essential Nutrition Action
FVS: Food variety score
GRT: Group- or cluster-randomized trials
HAZ: Height-for-age *z*-score
Hb: Hemoglobin concentration
HDSS: Health and Demographic Surveillance System
HFIAS: Household Food Insecurity Access Scale
HHS: Household Hunger Scale
HIGH: Heidelberg Institute of Global Health
HIV: Human immunodeficiency virus
HKI: Helen Keller International
ICC: Intra-class correlation coefficient
IPCC: Intergovernmental Panel on Climate Change
IQR: Interquartile range
IYCF: Infant and Young Child Feeding
KEMRI: Kenya Medical Research Institute
LMICs: Low- and middle-income countries
MAD: Minimum acceptable diet
MAR: Mean adequacy ratios
MDD: Minimum dietary diversity
MMF: Minimum meal frequency
N_2_O: Nitrous oxide
NGO: Non-governmental organization
NIH: National Institutes of Health
PCA: Principal component analysis
PI: Principal investigator
ppm: Parts per million
PPS: Probability proportional to population size
SD: Standard deviation
SERU: Scientific and Ethics Review Unit
SOPs: Standard Operating Procedures
WAZ: Weight-for-age *z*-score
WBCs: White blood cells
WHZ: Weight-for-height *z*-score
WHO: World Health Organization
