# Correction: ALIMUS—We are feeding! Study protocol of a multi-center, cluster-randomized controlled trial on the effects of a home garden and nutrition counseling intervention to reduce child undernutrition in rural Burkina Faso and Kenya

**DOI:** 10.1186/s13063-022-06450-2

**Published:** 2022-06-13

**Authors:** Isabel Mank, Raissa Sorgho, Fanta Zerbo, Moubassira Kagoné, Boubacar Coulibaly, John Oguso, Michael Mbata, Sammy Khagayi, Erick M. O. Muok, Ali Sié, Ina Danquah

**Affiliations:** 1 Heidelberg Institute of Global Health (HIGH), Medical Faculty and University Hospital Heidelberg, Heidelberg University, Im Neuenheimer Feld 324, 69120 Heidelberg, Germany; 2 German Institute for Development Evaluation (DEval), Bonn, Germany; 3 Centre de Recherche en Santé de Nouna (CRSN), Nouna, Burkina Faso; 4 Kenya Medical Research Institute (KEMRI), Centre for Global Health Research (CGHR), Kisumu, Kenya

**Correction: Trials 22:782 (2022)**

**https://doi.org/10.1186/s13063-022-06423-5**

Following the publication of the original article [1], we were notified that Figures 1 and 2 were incorrect in the pdf version. The XML version contained no mistakes.

The original article has been corrected.
